# Supplementary material for: Unravelling the history of hepatitis B virus genotypes A and D infection using a full-genome phylogenetic and phylogeographic approach
Source: eLife. 2018 Aug 7;7:e36709. doi: 10.7554/eLife.36709 (PMC6118819; doi:10.7554/eLife.36709)
Supplement: Supplementary file 1. [file elife-36709-supp1.docx]

**Supplementary Table 1.** Sampling of HBV^*^ genotype D sequences from different countries and geographic regions

| Country of sampling | Countries (N) | Sequences (N, %) | Monophyletic clusters (N) | Clustered sequences (N, %) |
| --- | --- | --- | --- | --- |
| Greenland | 1 | 10 **(1.1)** | 1 | 10 (100) |
| New Zealand | 1 | 37 **(4.0)** | 1 | 36 (97) |
| Japan | 1 | 36 **(3.9)** | 1 | 30 (83) |
| Tunisia | 1 | 50 **(5.5)** | 2 | 33 (66) |
| China | 1 | 31 **(3.4)** | 1 | 20 (65) |
| Belgium | 1 | 27 **(2.9)** | 1 | 6 (22) |
| India | 1 | **144 (15.7)** | **3** | **32 (22)** |
| Lebanon | 1 | 42 **(4.6)** | 1 | 7 (17) |
| Turkey | 1 | 97 **(10.6)** | 1 | 5 (5) |
| Iran | 1 | 135 **(14.7)** | 1 | 5 (4) |
| Russia | 1 | 20 **(2.2)** | 0 | 0 (0) |
| Syria | 1 | 70 **(7.7)** | 0 | 0 (0) |
| Region of sampling |  |  |  |  |
| Australasia and Oceania | 7 | 26 **(2.8)** | 1 | 19 (73) |
| Latin America and Caribbean | 4 | 36 **(3.9)** | 2 | 21 (58) |
| South, East and Southeast Asia | 4 | 21 **(2.3)** | 2 | 12 (57) |
| North America | 2 | 16 **(1.8)** | 1 | 8 (50) |
| Sub-Saharan Africa | 8 | 20 **(2.2)** | 2 | 10 (50) |
| Eastern Europe | 3 | 27 **(3.0)** | 1 | 5 (19) |
| Western Europe | 7 | **33 (3.6)** | **1** | **5 (15)** |
| Central Asia | 3 | 24 **(2.6)** | 0 | 0 (0) |
| Central Europe | 2 | 14 **(1.5)** | 0 | 0 (0) |
| Total | **52** | **916 (100)** | **23** | **264 (29)** |

^*^ HBV, *hepatitis B virus*
