# Supplementary material for: Unravelling the history of hepatitis B virus genotypes A and D infection using a full-genome phylogenetic and phylogeographic approach
Source: eLife. 2018 Aug 7;7:e36709. doi: 10.7554/eLife.36709 (PMC6118819; doi:10.7554/eLife.36709)
Supplement: Supplementary file 2. [file elife-36709-supp2.docx]

**Supplementary Table 2.** Sampling of HBV^*^ genotype A sequences from different countries and geographic regions

| Country of sampling | Countries (N) | Sequences (N, %) | Monophyletic clusters (N) | Clustered sequences (N, %) |
| --- | --- | --- | --- | --- |
| Haiti | 1 | 47 **(9.5)** | 3 | 44 (94) |
| Brazil | 1 | 24 **(4.8)** | 2 | 20 (83) |
| Panama | 1 | 17 **(3.4)** | 1 | 13 (76) |
| South Africa | 1 | 62 **(12.6)** | 3 | 43 (69) |
| Cameroon | 1 | 16 **(3.3)** | 1 | 8 (50) |
| Belgium | 1 | 78 **(15.8)** | 1 | 33 (42) |
| Argentina | 1 | 19 **(3.9)** | 1 | 6 (32) |
| Japan | 1 | 53 **(10.8)** | 2 | 11 (21) |
| India | 1 | 19 **(3.9)** | 0 | 0 (0) |
| France | 1 | 17 **(3.5)** | 0 | 0 (0) |
| Poland | 1 | 35 **(7.1)** | 0 | 0 (0) |
| USA | 1 | **10 (2.0)** | **0** | **0 (0)** |
| Region of sampling |  |  |  |  |
| Caribbean | 1 | 5 **(1.0)** | 0 | 0 (0) |
| Central Asia | 1 | 1 **(0.2)** | 0 | 0 (0) |
| Central Europe | 1 | 2 **(0.4)** | 0 | 0 (0) |
| East Asia | 2 | 4 **(0.8)** | 0 | 0 (0) |
| Eastern Europe | 4 | 17 **(3.5)** | 0 | 0 (0) |
| Latin America | 2 | 2 **(0.4)** | 0 | 0 (0) |
| North America | 1 | 1 **(0.2)** | 0 | 0 (0) |
| North Africa/Middle East | 2 | 2 **(0.4)** | 0 | 0 (0) |
| South Asia | 2 | 7 **(1.4)** | 0 | 0 (0) |
| Southeast Asia | **2** | **8 (1.6)** | 0 | 0 (0) |
| Sub-Saharan Africa | **12** | **34 (6.9)** | 0 | 0 (0) |
| Western Europe | 6 | **13 (2.6)** | **0** | **0 (0)** |
| Total | **48** | **493 (100)** | **14** | **178 (36)** |

^*^ HBV, *hepatitis B virus*
