# Supplementary material for: Unravelling the history of hepatitis B virus genotypes A and D infection using a full-genome phylogenetic and phylogeographic approach
Source: eLife. 2018 Aug 7;7:e36709. doi: 10.7554/eLife.36709 (PMC6118819; doi:10.7554/eLife.36709)
Supplement: Supplementary file 3. [file elife-36709-supp3.docx]

| **Supplementary Table 3:** List of papers for HBV* genotype D sequences included in the analysis | |
| --- | --- |
| **PMID** | **Title** |
| 1923778 | Sequence analysis of hepatitis B virus genome of a new mutant of ayw subtype isolated in Sardinia. |
| 2345966 | Active hepatitis B virus replication in the presence of anti-HBe is associated with viral variants containing an inactive pre-C region. |
| 3171552 | Typing hepatitis B virus by homology in nucleotide sequence: comparison of surface antigen subtypes. |
| 7489966 | Fulminant hepatitis associated with hepatitis B virus e antigen-negative infection: importance of host factors. |
| 8107226 | Enhanced replication of a hepatitis B virus mutant associated with an epidemic of fulminant hepatitis. |
| 8257295 | Sequence analysis of hepatitis B virus DNA in immunologically negative infection. |
| 8871878 | Whole genome analysis of hepatitis B virus from four cases of fulminant hepatitis: genetic variability and its potential role in disease pathogenicity. |
| 9188611 | Transcription of hepatitis B virus in peripheral blood mononuclear cells from persistently infected patients. |
| 9581787 | Analysis of hepatitis B virus populations in an interferon-alpha-treated patient reveals predominant mutations in the C-gene and changing e-antigenicity. |
| 10223539 | Infection chains and evolution rates of hepatitis B virus in cardiac transplant recipients infected nosocomially. |
| 10534721 | Hepatitis B virus core promoter mutations in children with multiple anti-HBe/HBeAg reactivations result in enhanced promoter activity. |
| 10640544 | Long-term mutation rates in the hepatitis B virus genome. |
| 11257194 | A novel variant genotype C of hepatitis B virus identified in isolates from Australian Aborigines: complete genome sequence and phylogenetic relatedness. |
| 11966946 | Profile, spectrum and significance of HBV genotypes in chronic liver disease patients in the Indian subcontinent. |
| 12124470 | Genotype H: a new Amerindian genotype of hepatitis B virus revealed in Central America. |
| 12480564 | Complete genome sequence of hepatitis B virus (HBV) from a patient with fulminant hepatitis without precore and core promoter mutations: comparison with HBV from a patient with acute hepatitis infected from the same infectious source. |
| 14981757 | Comparison of genotypes C and D of the hepatitis B virus in Japan: a clinical and molecular biological study. |
| 15105537 | Distinctive sequence characteristics of subgenotype A1 isolates of hepatitis B virus from South Africa. |
| 15184493 | Hepatitis B surface antigen variant with multiple mutations in the a determinant in an agammaglobulinemic patient. |
| 15332270 | Hepatitis B virus genotype D strains from Estonia share sequence similarity with strains from Siberia and may specify ayw4. |
| 15382123 | Factors associated with fulminant liver failure during an outbreak among injection drug users with acute hepatitis B. |
| 15902699 | Complete genomic sequence and phylogenetic relatedness of hepatitis B virus isolates from Iran. |
| 15977237 | Complete genome sequence and phylogenetic analysis of hepatitis B virus isolated from Turkish patients with chronic HBV infection. |
| 16299716 | Tracing the history of hepatitis B virus genotype D in western Japan. |
| 16847957 | Phylogenetic relatedness and genetic diversity of hepatitis B virus isolates in Eastern India. |
| 16991006 | Complete genome sequence and phylogenetic analysis of hepatitis B virus (HBV) isolated from Mongolian patients with chronic HBV infection. |
| 17006908 | Influence of hepatitis B virus genotypes on the intra- and extracellular expression of viral DNA and antigens. |
| 17191017 | Heterogeneity of hepatitis B virus genotype D in Japan. |
| 17397513 | Characteristics of geographic distributions and route of infection for hepatitis B virus genotype D in Ehime area in western Japan. |
| 18098129 | Hepatitis B virus transmission pattern and vaccination efficiency in Uzbekistan. |
| 18318825 | Virological and clinical implication of core promoter C1752/V1753 and T1764/G1766 mutations in hepatitis B virus genotype D infection in Mongolia. |
| 18373690 | Multiple genotypes and subtypes of hepatitis B and C viruses in Belarus: similarities with Russia and western European influences. |
| 18551607 | Multiple surface antigen mutations in five blood donors with occult hepatitis B virus infection. |
| 18632953 | D2: major subgenotype of hepatitis B virus in Russia and the Baltic region. |
| 18649329 | Molecular characterization of hepatitis B virus (HBV) isolates, including identification of a novel recombinant, in patients with acute HBV infection attending an Irish hospital. |
| 18952249 | Analysis of hepatitis B virus X gene phylogeny, genetic variability and its impact on pathogenesis: implications in Eastern Indian HBV carriers. |
| 18985816 | Identification and characterization of genotype A and D recombinant hepatitis B virus from Indian chronic HBV isolates. |
| 19339480 | A novel hepatitis B virus subgenotype, D7, in Tunisian blood donors. |
| 19386834 | Complete genome sequence and phylogenetic relatedness of hepatitis B virus isolates in Papua, Indonesia. |
| 19535503 | Deletions and recombinations in the core region of hepatitis B virus genotype E strains from asymptomatic blood donors in Guinea, west Africa. |
| 19691824 | Hepatitis B virus genotypes/subgenotypes in voluntary blood donors in Makassar, South Sulawesi, Indonesia. |
| 19751583 | Slave trade and hepatitis B virus genotypes and subgenotypes in Haiti and Africa. |
| 19780948 | Molecular and serological characterization of hepatitis B virus genotype A and D infected blood donors in Poland. |
| 20087936 | Molecular evolutionary analysis and mutational pattern of full-length genomes of hepatitis B virus isolated from Belgian patients with different clinical manifestations. |
| 20147517 | A novel hepatitis B virus (HBV) subgenotype D (D8) strain, resulting from recombination between genotypes D and E, is circulating in Niger along with HBV/E strains. |
| 20174575 | Molecular and phylogenetic analyses suggest an additional hepatitis B virus genotype "I". |
| 20196797 | Diversity of hepatitis B virus infecting Malaysian candidate blood donors is driven by viral and host factors. |
| 20580309 | Acute hepatitis B infection associated with drug-resistant hepatitis B virus. |
| 20723037 | Distinct geographical and demographic distribution of hepatitis B virus genotypes in the Canadian Arctic as revealed through an extensive molecular epidemiological survey. |
| 20848146 | Association of hepatitis B virus mutations in basal core promoter and precore regions with severity of liver disease: an investigation of 793 Chinese patients with mild and severe chronic hepatitis B and acute-on-chronic liver failure. |
| 21108339 | Analysis of the full-length genomes of novel hepatitis B virus subgenotypes C11 and C12 in Papua, Indonesia. |
| 21108697 | An outbreak of hepatitis B with high mortality in India: association with precore, basal core promoter mutants and improperly sterilized syringes. |
| 21318309 | Ethnogeographical structure of hepatitis B virus genotype distribution in Indonesia and discovery of a new subgenotype, B9. |
| 21494570 | Geographical and ethnic distribution of the HBV C/D recombinant on the Qinghai-Tibet Plateau. |
| 21503905 | Full genome characterization of hepatitis B virus strains from blood donors in Iran. |
| 21601012 | Molecular characterization and phylogenetic analysis of full-genome HBV subgenotype D3 sequences from Serbia. |
| 21678435 | Characterization of hepatitis B virus in Turkish blood donors, and the prevalence of the SP1 splice variant. |
| 21765983 | Genomic analysis of hepatitis B virus reveals antigen state and genotype as sources of evolutionary rate variation. |
| 22246826 | Characterization of hepatitis B virus genome variability in Iranian patients with chronic infection, a nationwide study. |
| 22610589 | HBV/D1: a major HBV subgenotype circulating in Uyghur patients with chronic HBV infection in Xinjiang, China. |
| 22785194 | Hepatitis B virus DNA splicing in Lebanese blood donors and genotype A to E strains: implications for hepatitis B virus DNA quantification and infectivity. |
| 22827722 | Tracking the naturally occurring mutations across the full-length genome of hepatitis B virus of genotype D in different phases of chronic e-antigen-negative infection. |
| 23103897 | Rethinking therapeutic decisions for hepatitis B infection in Syria: insights into molecular monitoring. |
| 23301547 | Genetic diversity of hepatitis B virus genotypes B6, D and F among circumpolar indigenous individuals. |
| 23865777 | Molecular characterization of hepatitis B virus in liver disease patients and asymptomatic carriers of the virus in Sudan. |
| 23884366 | African, Amerindian and European hepatitis B virus strains circulate on the Caribbean Island of Martinique. |
| 23969496 | Identification of optimal target gene regions for hepatitis B virus genotyping by DNA sequencing. |
| 24026673 | In vitro replication competence of a hepatitis B genotype D/A recombinant virus: dissimilar biological behaviour regarding its parental genotypes. |
| 24642137 | High prevalence of hepatitis B virus subgenotypes A1 and D4 in Maranhão state, Northeast Brazil. |
| 24792512 | Novel quasi-subgenotype D2 of hepatitis B virus identified in Taiwanese aborigines. |
| 25333524 | Novel point and combo-mutations in the genome of hepatitis B virus-genotype D: characterization and impact on liver disease progression to hepatocellular carcinoma. |
| * HBV, hepatitis B virus | |
