# Supplementary material for: Unravelling the history of hepatitis B virus genotypes A and D infection using a full-genome phylogenetic and phylogeographic approach
Source: eLife. 2018 Aug 7;7:e36709. doi: 10.7554/eLife.36709 (PMC6118819; doi:10.7554/eLife.36709)
Supplement: Supplementary file 4. [file elife-36709-supp4.docx]

| **Supplementary Table4:** List of papers for HBV* genotype A sequences included in the analysis | |
| --- | --- |
| **PMID** | **Title** |
| 9774595 | Distribution of hepatitis B virus genotypes in two different pediatric populations from Argentina. |
| 9930189 | Hepatitis B virus genomic seuence in the circulation of hepatocellular carcinoma patients: comparative analysis of 40 full-length isolates. |
| 10347133 | Three cases of severe subfulminant hepatitis in heart-transplanted patients after nosocomial transmission of a mutant hepatitis B virus. |
| 11596083 | Molecular analysis of hepatitis B virus genomes isolated from black African patients with fulminant hepatitis B. |
| 12436475 | Genotype, serotype, and phylogenetic characterization of the complete genome sequence of hepatitis B virus isolates from Malawian chronic carriers of the virus. |
| 12767980 | Genome replication, virion secretion, and e antigen expression of naturally occurring hepatitis B virus core promoter mutants. |
| 14662927 | Evaluation of the INNO-LiPA HBV genotyping assay for determination of hepatitis B virus genotype. |
| 15039524 | Epidemiological and sequence differences between two subtypes (Ae and Aa) of hepatitis B virus genotype A. |
| 15105537 | Distinctive sequence characteristics of subgenotype A1 isolates of hepatitis B virus from South Africa. |
| 15332270 | Hepatitis B virus genotype D strains from Estonia share sequence similarity with strains from Siberia and may specify ayw4. |
| 15958684 | A new subtype (subgenotype) Ac (A3) of hepatitis B virus and recombination between genotypes A and E in Cameroon. |
| 15962285 | Functional analysis of hepatitis B virus reactivating in hepatitis B surface antigen-negative individuals. |
| 16033963 | Phylogeny of African complete genomes reveals a West African genotype A subtype of hepatitis B virus and relatedness between Somali and Asian A1 sequences. |
| 16603517 | Phylogenetic analysis of the precore/core gene of hepatitis B virus genotypes E and A in West Africa: new subtypes, mixed infections and recombinations. |
| 16760389 | Novel subtypes (subgenotypes) of hepatitis B virus genotypes B and C among chronic liver disease patients in the Philippines. |
| 16847965 | Identification of hepatitis B virus subgenotype A3 in rural Gabon. |
| 16952546 | Functional analysis of complex hepatitis B virus variants associated with development of liver cirrhosis. |
| 17006908 | Influence of hepatitis B virus genotypes on the intra- and extracellular expression of viral DNA and antigens. |
| 17700028 | Immune response induced by a different combined immunization of HBsAg vaccine. |
| 18061229 | Co-replication analyses of naturally occurring defective hepatitis B virus variants with wild-type. |
| 18077651 | Prevalence and genetic diversity of hepatitis B and delta viruses in pregnant women in Gabon: molecular evidence that hepatitis delta virus clade 8 originates from and is endemic in central Africa. |
| 18086305 | HBVRegDB: annotation, comparison, detection and visualization of regulatory elements in hepatitis B virus sequences. |
| 18098129 | Hepatitis B virus transmission pattern and vaccination efficiency in Uzbekistan. |
| 18373690 | Multiple genotypes and subtypes of hepatitis B and C viruses in Belarus: similarities with Russia and western European influences. |
| 18479961 | Hepatitis B virus genetic diversity in Argentina: dissimilar genotype distribution in two different geographical regions; description of hepatitis B surface antigen variants. |
| 18507755 | Two simultaneous hepatitis B virus epidemics among injecting drug users and men who have sex with men in Buenos Aires, Argentina: characterization of the first D/A recombinant from the American continent. |
| 18606836 | Rolling circle amplification, a powerful tool for genetic and functional studies of complete hepatitis B virus genomes from low-level infections and for directly probing covalently closed circular DNA. |
| 18632953 | D2: major subgenotype of hepatitis B virus in Russia and the Baltic region. |
| 18985816 | Identification and characterization of genotype A and D recombinant hepatitis B virus from Indian chronic HBV isolates. |
| 19152415 | Exceptional genetic variability of hepatitis B virus indicates that Rwanda is east of an emerging African genotype E/A1 divide. |
| 19297602 | Distribution of hepatitis B virus genotypes among patients with chronic infection in Japan shifting toward an increase of genotype A. |
| 19439548 | Prevalence and molecular diversity of hepatitis B virus and hepatitis delta virus in urban and rural populations in northern Gabon in central Africa. |
| 19535503 | Deletions and recombinations in the core region of hepatitis B virus genotype E strains from asymptomatic blood donors in Guinea, west Africa. |
| 19615936 | Phylogenetic analysis of hepatitis B virus full-length genomes reveals evidence for a large nosocomial outbreak in Belgium. |
| 19751583 | Slave trade and hepatitis B virus genotypes and subgenotypes in Haiti and Africa. |
| 19780948 | Molecular and serological characterization of hepatitis B virus genotype A and D infected blood donors in Poland. |
| 19857994 | Novel hepatitis B virus subgenotype A6 in African-Belgian patients. |
| 20087936 | Molecular evolutionary analysis and mutational pattern of full-length genomes of hepatitis B virus isolated from Belgian patients with different clinical manifestations. |
| 20219082 | Detection of a new subgenotype of hepatitis B virus genotype A in Cameroon but not in neighbouring Nigeria. |
| 21181917 | Molecular characterization of hepatitis B virus isolates from Zimbabwean blood donors. |
| 21248087 | Outbreak of infections by hepatitis B virus genotype A and transmission of genetic drug resistance in patients coinfected with HIV-1 in Japan. |
| 21328372 | Combined use of wild-type HBV precore and high serum iron marker as a potential tool for the prediction of cirrhosis in chronic hepatitis B infection. |
| 21981386 | Hepatitis B virus transmission by blood transfusion during 4 years of individual-donation nucleic acid testing in South Africa: estimated and observed window period risk. |
| 22459735 | Probable corticosteroid-induced reactivation of latent hepatitis B virus infection in an HIV-positive patient involving immune escape. |
| 22510694 | Convergence and coevolution of hepatitis B virus drug resistance. |
| 23092209 | Phylogenetic analysis of complete genome sequences of hepatitis B virus from an Afro-Colombian community: presence of HBV F3/A1 recombinant strain. |
| 24197437 | Surface antigen-negative hepatitis B virus infection in Dutch blood donors. |
| 24681050 | Construction of replication competent plasmids of hepatitis B virus subgenotypes A1, A2 and D3 with authentic endogenous promoters. |
| 25122004 | Hepatitis B virus subgenotype A1: evolutionary relationships between Brazilian, African and Asian isolates. |
| 25149225 | Circulation of genotype-I hepatitis B virus in the primitive tribes of Arunachal Pradesh in early sixties and molecular evolution of genotype-I. |
| 25742179 | HBV genotypic variability in Cuba. |
| 25822666 | Hepatitis B virus genotype distribution and genotype-specific BCP/preCore substitutions in acute and chronic infections in Argentina. |
| 26100402 | Genetic variability of hepatitis B virus in Uruguay: D/F, A/F genotype recombinants. |
| 26230260 | Phylogenetic Analysis of Hepatitis B Virus Genotypes Circulating in Different Risk Groups of Panama, Evidence of the Introduction of Genotype A2 in the Country. |
| 26342803 | A rare case of HBV genotype fluctuation (shifting and reversion) after liver transplantation. |
| 26659668 | Complete Genome Sequences of Hepatitis B Virus from North India Using Ion Torrent. |
| 27002608 | Hepatitis B virus genotype A: design of reference sequences for sub-genotypes. |

* HBV, hepatitis B virus
