# Supplementary material for: Unravelling the history of hepatitis B virus genotypes A and D infection using a full-genome phylogenetic and phylogeographic approach
Source: eLife. 2018 Aug 7;7:e36709. doi: 10.7554/eLife.36709 (PMC6118819; doi:10.7554/eLife.36709)
Supplement: Supplementary file 5. [file elife-36709-supp5.docx]

| **Supplementary Table 5:** List of countries within each geographic region (as defined by the Global Burden of Disease classification system), in which HBV* genotype D sequences were included in the analysis | |
| --- | --- |
| **Geographic region** | **Countries of sampling** |
| Asia-Pacific | Japan |
| Austalasia | Austalia, New Zealand |
| Caribbean | Haiti, Martinique |
| Central Asia | Kazakstan, Uzmbekistan, Mongolia, Russia† |
| Central Europe | Serbia, Poland |
| East Asia | China, Taiwan |
| Eastern Europe | Latvia, Estonia, Belarus |
| Latin America | Brazil, Argentina |
| North Africa and the Middle East | Iran, Turkey, Syria, Lebanon, Tunisia |
| North America | Canada, the United States of America (USA) |
| Oceania | Samoa, Tonga, Fiji, Kiribati, New Caledonia, Papua New Guinea |
| South Asia | India, Pakistan |
| Southeast Asia | Indonesia, Malaysia |
| Sub-Saharan Africa | South Africa, Niger, Mauritius, the Central African Republic, Gabon, Ghana, Sudan, Kenya |
| Western Europe | Greece, Belgium, Italy, Germany, Spain, Ireland, France, Sweden, Greenland |
| † Russia according to the Global Burden of Disease classification system belongs to the region of “Eastern Europe”. In our study 16 of 20 (80%) sequences from Russia were sampled in the Asian part of the country and specifically at Khabarovsk, Chita, Krasnoyarsk, Yakutsk and Kemerovo. For this reason we classified Russia in the region of “Central Asia”.  * HBV, hepatitis B virus | |
