# Supplementary material for: Unravelling the history of hepatitis B virus genotypes A and D infection using a full-genome phylogenetic and phylogeographic approach
Source: eLife. 2018 Aug 7;7:e36709. doi: 10.7554/eLife.36709 (PMC6118819; doi:10.7554/eLife.36709)
Supplement: Supplementary file 6. [file elife-36709-supp6.docx]

| **Supplementary Table 6:** List of countries within each geographic region (as defined by the Global Burden of Disease classification system), in which HBV* genotype A sequences were included in the analysis | |
| --- | --- |
| **Geographic region** | **Countries of sampling** |
| Asia-Pacific | Japan |
| Caribbean | Haiti, Cuba |
| Central Asia | Uzmbekistan |
| Central Europe | Serbia, Poland |
| East Asia | China, Taiwan |
| Eastern Europe | Latvia, Estonia, Russia, Belarus |
| Latin America | Brazil, Argentina, Panama, Colombia, Uruguay |
| North Africa and the Middle East | Turkey, the United Arab Emirates |
| North America | Canada, the United States of America (USA) |
| South Asia | India, Bangladesh, Nepal |
| Southeast Asia | The Philippines, Malaysia |
| Sub-Saharan Africa | South Africa, Cameroon, Rwanda, Somalia, Gabon, Kenya, the Gambia, Malawi, Nigeria, Zimbabwe, Guinea, Tanzania, Uganda, the Central African Republic |
| Western Europe | Belgium, France, Germany, Italy, the Netherlands, Denmark, Spain, Sweden |

* HBV, hepatitis B virus
